# Assessing evidence of measurement invariance of the Mental Health Inventory (MHI-5) by gender in a German adult sample

**DOI:** 10.1186/s12888-025-07463-2

**Published:** 2025-10-02

**Authors:** Bianca Savic, Andreas Staudt, Anne Moehring, Diana Guertler, Hans-Juergen Rumpf, Ulrich John, Sophie Baumann

**Affiliations:** 1 Department of Methods in Community Medicine, Institute for Community Medicine, University Medicine Greifswald, Walther‑Rathenau‑Str. 48, Greifswald, 17475 Germany; 2 Faculty of Medicine, Technische Universität Dresden, Institute and Policlinic of Occupational and Social Medicine, Fetscherstr. 74, Dresden, 01307 Germany; 3 Department of Psychiatry and Psychotherapy, University of Lübeck, Ratzeburger Allee 160, Lübeck, 23538 Germany; 4 Department of Prevention Research and Social Medicine, Institute for Community Medicine, University Medicine Greifswald, Walther‑Rathenau‑Str. 48, Greifswald, 17475 Germany

**Keywords:** Measurement invariance, Mental Health Inventory-5, MHI-5, Gender, CFA, Psychometrics

## Abstract

**Background:**

The Mental Health Inventory-5 (MHI-5) is an internationally used screening measure for assessing mental health, with evidence supporting validity and reliability in different settings. So far, measurement invariance of the MHI-5, especially regarding gender, has rarely been investigated. Therefore, the aim of this study was to examine measurement invariance of the MHI-5 between men and women in a German population sample to enable valid and generalizable group comparisons.

**Methods:**

A total of 2075 participants (52.5% women) aged 18–64 years (M = 32.7, SD = 11.7) were proactively recruited at a municipal registry office in Germany and completed the MHI-5. The underlying factor structure was examined by comparing a one-factor model to a two-factor model. Multiple group confirmatory factor analysis was used to investigate measurement invariance between men and women.

**Results:**

A two-factor structure showed better model fit (χ^2^(4) = 106.388, Comparative Fit Index, CFI = 0.986) compared to a one-factor structure (χ^2^(5) = 193.652, CFI = 0.974). Constraining thresholds to equality did not lead to a significant loss of fit (χ^2^(10) = 9.370; *p* = .497, ΔCFI = .005). Further constraining loadings also did not reduce model fit (Δχ^2^(3) = 2.036; *p* = .565, ΔCFI = .001).

**Conclusion:**

The results demonstrated threshold and loading invariance for the MHI-5 between men and women. These findings suggest that the MHI-5 can be used for meaningful comparisons of latent means between men and women in the German population.

**Trial registration:**

German Clinical Trials Register (trial registration number: DRKS00014274, date of registration: 12 March 2018).

**Supplementary Information:**

The online version contains supplementary material available at 10.1186/s12888-025-07463-2.

## Background

Mental health is an important aspect of overall well-being and has been gaining increasing recognition as an essential public health matter [1]. Given its significance, questionnaires have become an important tool in the screening of mental health issues. They offer a cost-effective and standardized approach to assess mental health. There are numerous validated screening instruments, each capturing different psychological constructs, such as depression [2], anxiety [3], or overall psychological distress [4]. To provide a more efficient and comprehensive assessment of mental health, capturing both psychological distress and well-being, the five-item Mental Health Inventory (MHI-5) has been developed as a shortened version of the original 38-item MHI [5]. It consists of the five items that most accurately reflect the total score of the original version [6]. It assesses symptoms of both depression and anxiety but also psychological well-being over the past month, aligning with dual-factor concepts of mental health. Psychopathology and well-being are viewed as distinct but connected dimensions [7, 8]. This offers an advantage to other screening instruments, such as Patient Health Questionnaire-9 (PHQ-9) or Generalized Anxiety Disorder-7 (GAD-7), which assess one dimension of psychopathology. Another operational advantage of the MHI-5 is its inclusion in the SF-36, which enables its use not only in mental health surveys but also in general health surveys. As a result, it is frequently used in broader population-based studies and not limited to psychiatric patient samples [9, 10]. Psychometric properties of the MHI-5 have been examined in numerous studies, repeatedly supporting evidence of internal consistency and construct validity [4, 11–13] across various age groups [10, 14–16], different countries [10, 15, 16], and in different subpopulations [17, 18].

Sensitivity and specificity of the MHI-5 have been reported in multiple studies, generally demonstrating good screening accuracy. Sensitivity ranges from 73 to 91%, and specificity from 58 to 84%, depending on the population and cut-off used [19–21].

Multiple studies have used the MHI-5 as an outcome measure to assess mental health status across various populations and research contexts, including population-based screening studies [21, 22], longitudinal and cross-sectional studies [23–26], as well as intervention studies [27–30]. Moreover, the MHI-5 is often used to compare mental health across sociodemographic groups, such as gender. Gender differences in mental health are well-documented. Women consistently report higher levels of internalizing disorders, such as depression and anxiety [31–33], while men show higher rates of externalizing disorders, such as substance abuse [32, 34, 35]. These gender differences are attributed to biological, psychological, and social factors that influence the prevalence, expression, and reporting of mental health symptoms [36–38]. A systematic review of three large German population-based cohort studies reported significantly worse mental health in women than men in all cohorts [35, 39, 40]. Similarly, a German cross-sectional study among students found that mental health, as indicated by a subscale of the SF-36, was significantly lower among women [41]. Comparable findings using the MHI-5 were reported from population-based studies in Norway [9] and Finland [42]. As the MHI-5 assesses a latent construct of mental health, measuring non-observable variables, group comparisons based on mean scores require establishing measurement invariance to be established prior to use, in order to allow for meaningful comparisons. Measurement invariance means that the instrument measures the same underlying construct in a comparable way across different groups. This assures that any observed differences in scores truly reflect differences between mean scores rather than differences in how groups interpret or respond to the items [43–45]. Despite its importance, measurement invariance is often assumed without formally being tested before comparing means of latent constructs [46–48]. This oversight can lead to biased or misleading conclusions, as differences in scores may not reflect true group differences [49]. The conventional steps for testing measurement invariance include a series of increasingly restrictive models in the following order: First, configural invariance tests whether the overall factor structure is similar across groups. Next, metric or loading invariance tests if each item on the scale loads onto the latent variable in a similar manner [50]. Thirdly, scalar invariance indicates that the intercepts or thresholds of the items are equal, allowing for the comparison of latent means. Thresholds refer to the point at which a response transitions from one point to the next on an ordered-categorical scale. Lastly, strict invariance additionally indicates equal residual variances. It is rarely tested in practice as it is a highly constrained model [51]. While these sequential steps are standard for continuous variables, a different approach is recommended for categorical variables [52, 53]. Following the recommendations by Wu and Estabrook [52], first configural invariance is tested, followed by threshold invariance, and lastly, loading and threshold invariance. Regarding the MHI-5, to our knowledge, there has been one study examining measurement invariance across gender [54]. This study found no evidence of metric and scalar invariance of the Spanish version of the MHI-5 using a two-factor model. This indicates that the instrument may not measure the same construct equivalently across these groups, and therefore, comparisons between men and women may be biased. However, it is important to note that large sample sizes and psychometrically robust instruments increase the likelihood of detecting even minor non-invariance. Additionally, this study applied the conventional sequence of invariance testing, typically used for continuous data, rather than the alternative sequence recommended for categorical data, which may partly explain the observed non-invariance [52].

Therefore, the aim of our study was to examine measurement invariance among men and women for the MHI-5 in a German population, considering the categorical nature of the data, in order to ensure valid comparisons between these groups.

## Methods

This study was based on screening data from the randomized controlled trial entitled “Testing a proactive expert system intervention to prevent and to quit at-risk alcohol use” (PRINT) [55].

### Sample recruitment

From April to June 2018, participants were recruited at the registry office in Greifswald, Mecklenburg-West Pomerania, Germany. All persons aged 18–64 years appearing in the waiting area were approached by study assistants and asked to respond to an electronic self-administered lifestyle screening. Persons already approached during an earlier visit, with notable cognitive impairment or a physical condition that prevents participation, with insufficient German language or reading skills, or employed at the conducting research institute, were excluded.

As described in more detail elsewhere [56], 2947 out of 3969 eligible persons completed the screening assessment (74%), 879 declined (22%), and 143 (4%) were not screened for other reasons (e.g., not approached because waiting time was too short). Among those, 872 (30%) had to be excluded from the current analysis because the tablet computers accidentally omitted the MHI-5 questions in the course of the first two weeks of recruitment. After the technical problem was noticed and fixed, the assessment yielded 2075 participants who completed the MHI-5 and were used for the current analysis. Although the data loss occurred systematically for all participants in the starting phase of the recruitment period, those with missing data on the MHI-5 were more likely to be female (χ^2^ (1) = 8.44, *p* = 0.004) and younger (*t* (2945) = −2.72, *p* = 0.007). This is most probably due to a higher proportion of university students consulting the registry office during the first two weeks of recruitment compared to the remaining period (χ^2^ (1) = 13.23, *p* < 0.001).

### Measures

The MHI-5 is a brief self-reported measure used frequently in clinical and research settings to assess mental health. The German version of the MHI-5 [12], with a five-point Likert-type scale instead of the original six-point scale, was used. It is part of the widely used SF-36 questionnaire and a short version of the original 38-item MHI [5]. The MHI-5 contains the following questions: “How much of the time during the last month have you (Item 1) been a very nervous person?, (Item 2) felt calm and peaceful?, (Item 3) felt downhearted and blue?, (Item 4) been a happy person?, and (Item 5) felt so down in the dumps that nothing could cheer you up?” and a five-point Likert-type scale ranging from 1 (“none of the time “) to 5 (“all of the time”). Items 1, 3, and 5 were reverse-coded. The sum score was standardized by linear transformation, with the scale ranging from 0 to 100, and higher scores indicating better mental health. Previous studies have suggested different cut-off values, ranging from ≤ 65 [12] to ≤ 68 [57] and up to ≤ 72 in other studies [22], depending on the population and research question. Gender was assessed by the question “Are you…”, with answer choices being “man” and “woman”. Other socio-demographic variables included age (in years), partnership (yes/no), educational background, and employment status. Participants indicated their highest general educational degree. The educational background was assessed by asking participants to indicate their highest general educational degree. The response options included an exhaustive list of possible school-leaving qualifications in Germany and equivalent foreign degrees. The information was categorized into three groups: < 10 years of school education, 10–11 years of school education, and ≥ 12 years of school education. Employment status encompassed full-time employed, part-time employed, in education, unemployed, and other (e.g., retired). Self-rated health was assessed with the question „Would you say your health in general is: excellent, very good, good, fair, or poor? “ [58]. Fair and poor were merged due to the limited number of participants in each of the two categories. Behavior-related variables included the alcohol consumption as indicated by the Alcohol Use Disorders Identification Test – Consumption (AUDIT-C) sum score [59], the number of cigarettes per day, and the body mass index obtained from self-reported body weight in kilograms and body height in meters, and the formula weight divided by height squared.

### Statistical analysis

To examine measurement invariance between men and women, we conducted a series of multi-group confirmatory factor analyses using Mplus Version 8.8 Demo for Mac OS X (Muthén & Muthén, 2017). We specified the data as categorical and used the mean- and variance-adjusted weighted least squares (WLSMV) estimator [60]. Scaled values of the Comparative Fit Index (CFI) and Tucker-Lewis Index (TLI) ≥ 0.90 [61] as well as values of the Standardized Root Means Squared Residual (SRMR) ≤ 0.08 and the Root Mean Square Error of Approximation (RMSEA) ≤ 0.08 [62, 63] were used as indicators of acceptable model fit. We examined the underlying factor structure by comparing a one-factor model to a two-factor model in terms of model fit. To test for measurement invariance, a series of increasingly restrictive models were estimated following the procedure recommended for categorical data by Svetina et. al [53]. These steps differ from the typical sequence used for continuous data, which generally involves testing for configural, metric (loading), scalar (intercept or threshold), and strict invariance in that order. In contrast, for categorical data, threshold invariance is tested prior to constraining factor loadings [52].

First, a CFA model was estimated separately for men and women, followed by a multi-group baseline model without any equality constraints. An acceptable model fit for each group, as well as the baseline model, would indicate configural invariance. In a second step, a threshold invariance model was fitted by constraining thresholds to be equal across gender. For model identification purposes, specific parameters were fixed, as recommended by Wu and Estabrook [52] (Table 1). If threshold invariance was supported, then a combined loading and threshold model was fitted in a third step by additionally constraining factor loadings to equality. If invariance was not supported at any of the steps described above, partial invariance was tested by iteratively freeing the parameter constraints that had the most significant contribution to misfit, according to modification indices. To evaluate measurement invariance, each model was compared sequentially to the next, more constricted model using chi-square (χ^2^) difference tests to determine if the additional constraints significantly decreased the model fit. A *p*-value < 0.05 was considered to be statistically significant. In addition to that, differences in CFI, RMSEA, and SRMR values were reported. Cut-off values recommended by Chen [64] for sample sizes n > 300 were used: threshold non-invariance if ΔCFI ≥ −0.010 and ΔRMSEA ≥ 0.015 or ΔSRMR ≥ 0.030; combined threshold and loading non-invariance if ΔCFI ≥ −0.010 and ΔRMSEA ≥ 0.015 or ΔSRMR ≥ 0.010. Given the categorical nature of the data, model fit changes were interpreted alongside χ^2^-difference testing, as recommended [52, 53, 65].

**Table 1:** Steps for testing measurement invariance with categorical data

Model	Baseline Model	Threshold invariance model	Threshold and Loading invariance model
Factor means	Fixed at 0	Fixed at 0	Fixed at 0
Factor variances	Fixed at 1	Fixed at 1	Fixed at 1/*
Factor scale	Fixed at 1	Fixed at 1/*	Fixed at 1/*
Intercept means	Fixed at 0	Fixed at 0/*	Fixed at 1/*
Residual variances	Fixed at 0	Fixed at 0	Fixed at 0
Thresholds	*	Equal	Equal
Factor Loadings	*	*	Equal

‘*’ Indicates freely estimated parameters in both groups, ‘Equal’ means the parameter is equal in both groups, ‘Fixed at 1/*’ or ‘Fixed at 0/*’ indicates the parameter is fixed at 1/or 0 in the reference group, while freely estimated in the alternative group; ‘Fixed at 1’ or ‘Fixed at 0’ means the parameter is fixed at 1/0 in both groups

## Results

### Sample

As shown in Table 2, the final sample consisted of 1090 (52.5%) women and 985 (47.5%) men with a mean age of 32.7 years (SD = 11.7). The transformed mean standardized MHI-5 score for the total sample was 66.0 (SD = 16.0), with women reporting a mean score of 64.2 (SD = 16.2) and men 68.1 (SD = 15.6). The mean scores for each item on the five-point Likert scale can be found in Table 3.

**Table 2:** Baseline characteristics for the study sample

	**Total sample (*N* = 2075)**	**Women (*N* = 1090)**	**Men (*N* = 985)**
Standardized MHI-5 score	66.0 (16.0)	64.2 (16.2)	68.1 (15.6)
Age (years)	32.7 (11.7)	32.5 (11.7)	32.9 (11.7)
Education			
< 10 years	147 (7.1%)	67 (6.1%)	80 (8.1%)
10–11 years	653 (31.5%)	347 (31.8%)	306 (31.1%)
≥ 12 years	1275 (61.4%)	676 (62.0%)	599 (60.8%)
Employment status			
Full-time employed	852 (41.1%)	378 (34.7%)	474 (48.1%)
Part-time employed	422 (20.3%)	266 (24.4%)	156 (15.8%)
Education	448 (21.6%)	261 (23.9%)	187 (19.0%)
Unemployed	74 (3.6%)	27 (2.5%)	47 (4.8%)
Other	279 (13.4%)	158 (14.5%)	121 (12.3%)
Having a partner (yes)	1244 (60.0%)	659 (60.5%)	585 (59.4%)
Self-rated health			
Excellent	161 (7.8%)	72 (6.6%)	89 (9.0%)
Very good	767 (37.0%)	363 (33.3%)	404 (41.0%)
Good	978 (47.1%)	560 (71.4%)	418 (42.4%)
Fair/poor	169 (8.1%)	95 (8.7%)	74 (7.5%)
AUDIT-C score	3.0 (2.2)	2.7 (1.9)	3.4 (2.3)
No. cigarettes/day	3.4 (6.6)	2.9 (6.0)	3.8 (7.1)
Body mass index	24.7 (4.8)	24.1 (5.1)	25.4 (4.2)

Data are means (SD) or numbers (%)

*MHI-5* Mental Health Inventory—5, *AUDIT-C* Alcohol Use Disorders Identification Test-Consumption

**Table 3:** Means for each item on the five-point likert scale

	**Total sample (*N* = 2075)**	**Women (*N* = 1090)**	**Men (*N* = 985)**
Item 1: … been a very nervous person?	2.61 (.895)	2.75 (.891)	2.48 (.881)
Item 2: … felt calm and peaceful?	3.42 (.831)	3.37 (.829)	3.47 (.830)
Item 3: … felt downhearted and blue?	2.38 (.921)	2.54 (.905)	2.21 (.909)
Item 4: … been a happy person?	3.45 (.831)	3.46 (.829)	3.44 (.834)
Item 5: … felt so down in the dumps that nothing could cheer you up?	1.67 (.875)	1.71 (.894)	1.62 (.850)

Means (SD), items are not reverse-coded

### Measurement invariance analysis

#### Factor structure

First, we compared a one-factor model to a two-factor model (Table 4).

**Table 4:** Model fit one vs. two- factor model

Model fit	χ^2^ (df)	CFI	TLI	RMSEA	SRMR
One-factor model, without grouping	193.652 (5)	.974	.948	.135	.028
Two-factor model, without grouping	106.388 (4)	.986	.964	.111	.019

*CFI* Comparative Fit Index, *TLI* Tucker-Lewis Index, *RMSEA* Root Mean Square Error of Approximation, *SRMR* Standardized Root Means Squared Residual

The two-factor model showed improved fit indices compared to the one-factor model, indicating better representation of the latent construct.

Therefore, the following analyses were conducted using the two-factor model.

**Fig. 1:**
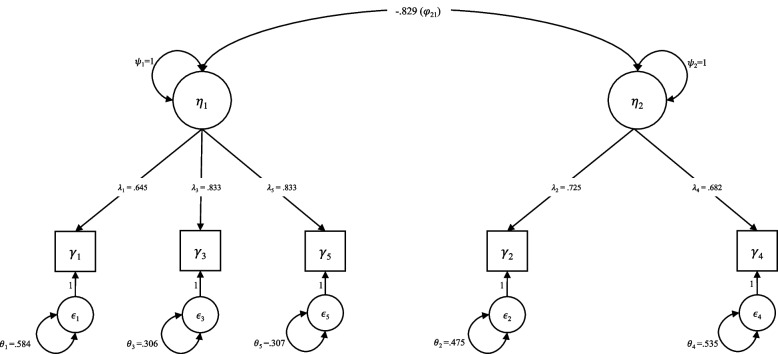
CFA path diagram. η_1_ = latent variable, psychological distress, η_2_ = latent variable, psychological well-being; ψ_1,2_ = latent factor variance, φ_21_ = covariance latent factors, λ_1–5_ = factor loadings, γ_1–5_ = observed indicators, ϵ_1–5_ = error terms, θ_1–5_ = error variances

Modification indices > 10 indicated potential local dependence between several item pairs, including Item 1 with Items 2 and 5 as well as Items 2 and 3. These pairs reflect either opposite poles of affective states or different facets of psychological distress, potentially resulting in residual correlations beyond the latent factors. Such correlations are not surprising in short scales assessing complex constructs and may reflect overlapping content or consistent response tendencies. However, no model modifications were made, as overall model fit was excellent and the original factor structure was retained based on theoretical considerations. Moreover, the standardized factor loadings (λ_1_ = 0.645, λ_2_ = 0.725, λ_3_ = 0.833, λ_4_ = 0.682, λ_5_ = 0.833) indicated a strong relationship between the observed variable and the underlying latent factor (Fig. 1).

#### Measurement invariance between men and women

The single-group CFA models showed acceptable fit indices for both women (χ^2^ = 65.890, *p* < 0.001; CFI = 0.985; TLI = 0.963; RMSEA = 0.119; SRMR = 0.021) and men (χ^2^ = 45.778, *p* < 0.001; CFI = 0.986; TLI = 0.965; RMSEA = 0.103; SRMR = 0.018) as well as the baseline multiple-group CFA model (χ^2^ = 111.864, *p* < 0.001; CFI = 0.985; TLI = 0.964; RMSEA = 0.112; SRMR = 0.020), suggesting configural invariance.

After restricting the thresholds to equality, the fit indices still indicated an acceptable fit, with improved CFI and RMSEA, and equal SRMR compared to the baseline model (Table 5). The χ^2^—difference testing was not significant, (Δχ^2^(10) = 9.370; *p* = 0.4974), suggesting threshold invariance. Furthermore, the differences in relative model fit indices were below the recommended cut-off value (ΔCFI = 0.005, ΔRMSEA = −0.049, ΔSRMR = 0.000), supporting this finding. Next, factor loadings were additionally constrained to be equal, in order to assess threshold and loading invariance. This model also demonstrated an acceptable fit, with further improved fit indices. The χ^2^-difference testing was non-significant (Δχ^2^(3) = 2.036; *p* = 0.5649) and differences in relative model fit indices were below the recommended cut-off value (ΔCFI = 0.001, ΔRMSEA = 0.010, ΔSRMR = 0.001), providing evidence of threshold and loading invariance.

**Table 5:** Model fit and difference testing

Model fit	χ^2^ (df)	p	CFI	TLI	RMSEA	SRMR
M1 Baseline model	111.864 (8)	<.001	.985	.964	.112	.020
M2: Thresholds constricted	91.864 (18)	<.001	.990	.989	.063	.020
M3: Thresholds and Loadings constricted	82.046 (21)	<.001	.991	.992	.053	.021
Difference Testing	Δχ^2^ (df)	p	ΔCFI		ΔRMSEA	ΔSRMR
Threshold invariance (M1 vs. M2)	9.370 (10)	.4974	.005		-.049	.000
Threshold and loading invariance (M2 vs. M3)	2.036 (3)	.5649	.001		.010	.001

*CFI* Comparative Fit Index, *TLI* Tucker-Lewis Index, *RMSEA* Root Mean Square Error of Approximation, *SRMR* Standardized Root Means Squared Residual

## Discussion

The MHI-5 is a widely used questionnaire to screen for mental health in research settings as well as in primary care or public health contexts. It has been found to be a valid and reliable measure across various populations [6, 12, 66]. The current work aimed to investigate measurement invariance of the MHI-5 between men and women in a German population-based sample to ensure validity of cross-group comparisons. We tested both one- and two-factor solutions to examine the underlying structure of the MHI-5. Although the original instrument has been treated as unidimensional in several studies [6, 17], recent studies support a bidimensional factor structure of the MHI-5 [15, 21, 54]. In our study, the two-factor model showed better model fit than the one-factor solution. This two-factor solution also fits well with the dual-factor model of mental health [7, 8], which views well-being and distress as related but distinct dimensions. The model fit indices generally supported the proposed model structure, although the RMSEA values exceeded conventional cut-off values for acceptable model fit. However, this should be interpreted with caution, as RMSEA tends to overestimate lack of model fit in models with few degrees of freedom and small number of items [67, 68]. Our data supported configural, threshold, and combined loading and threshold invariance. Configural invariance suggests that the MHI-5 has the same overall pattern of free and fixed factors for both groups [43, 44]. Threshold invariance suggests that response categories are interpreted equivalently across groups, ensuring that the latent construct's underlying scale is comparable [52, 53]. Establishing loading and threshold invariance allows for comparisons of latent means across groups as it confirms that the measurement scale and the strength of the relationship between items and the latent construct are equivalent [49, 52, 64, 69–71]. Overall, our results indicate that the MHI-5 measures the same latent construct between men and women within our German population sample, allowing for valid comparisons of latent mean scores. These findings contrast with Vilca et al.’s study [54], which used a Spanish version of the MHI-5. They found no evidence to support metric or scalar invariance, indicating that group differences may be due to the instrument functioning differently across gender rather than differences in mental health. Several factors may explain the different results. Although both studies used a two-factor model, differences in language version, sample composition, and cultural context may have influenced responses. Moreover, Vilca et al. applied the conventional testing sequence for measurement invariance, whereas we followed the adapted steps for categorical data, suggested by Wu and Estabrook [52]. Understanding whether the MHI-5 functions equivalently across gender is particularly important given the well-documented differences in mental health between men and women. It is widely acknowledged that the prevalence rate of depression and anxiety disorders tends to be higher among women [33, 72, 73]. In contrast to internalizing symptoms typically reported by women, men often present different symptoms of depression, including externalizing behavior such as anger, irritability, or substance abuse. These symptoms are less likely to be captured by conventional self-report tools and therefore may lead to underdiagnosis and undertreatment of depression and anxiety in men [72–74]. Additionally, mental health remains particularly stigmatized among men, and traditional masculine norms often prevent emotional expression and help-seeking [75, 76]. In this context, establishing measurement invariance before conducting group comparisons is essential to ensure that observed score differences reflect actual differences in the underlying construct. Failing to establish measurement invariance may lead to biased or misleading conclusions [43, 46, 47]. Aligning with our findings, similar results have been reported for other mental health screening instruments, such as the PHQ-9 or GAD-7, which also provided evidence of measurement invariance across gender [77–80]. While these results suggest that brief self-reported screening tools can provide comparable assessments of mental health, it is essential to acknowledge that gender-specific symptom expression and help-seeking behavior may still influence how individuals interpret or respond to specific items. Therefore, future research should investigate whether the inclusion of gender-specific items or subscales improves sensitivity and diagnostic accuracy of mental health screening instruments [81–83].

While this study provides valuable insights, there are some limitations that may impact the interpretation of the findings. First, although nearly three-fourths of the targeted population was reached through the proactive recruitment approach via the municipal registry office, selection bias cannot be ruled out. Our sample included a high number of persons with a high socio-economic position (SEP). Persons with higher SEP may more frequently make use of registry office services compared to those with lower SEP, e.g., registration of a new car. Another contributing factor may be the socio-demographic characteristics of the Greifswald population, which includes a large proportion of university employees and students. Relatedly, this study was conducted in one city in Germany, which may limit the generalizability of the findings to other regions or cultural contexts. Regional differences in socioeconomic conditions and cultural or ethnic subgroups within Germany may influence the way individuals interpret and respond to self-report instruments. Therefore, these results should be regarded with caution when applied beyond this specific German context.

Additionally, measurement invariance was tested across gender based on self-reported binary categories. It remains unclear whether participants interpreted this item regarding gender identity or their biological sex. While binary gender measures remain common in psychological research, they may exclude transgender, non-binary, or gender-diverse individuals. Future research should implement more inclusive gender measures to enable a more comprehensive understanding of how mental health instruments function across diverse gender groups.

## Conclusion

In summary, this study aimed to assess the validity of group comparisons by testing measurement invariance of the MHI-5 between men and women. Evidence of measurement invariance was found, supporting the conclusion that the MHI-5 functions equivalently across gender within this German population sample. The lack of previous research on measurement invariance of the MHI-5, especially across gender, highlights the importance of the present study. By addressing this gap, our findings contribute to a better understanding of the instrument’s validity of cross-group comparisons. Given its widespread international use, future research should build on these findings and investigate measurement invariance across other languages, regions, and cultural contexts. Additionally, future research should incorporate more inclusive assessments of gender identity to examine whether instruments like the MHI-5 function equivalently across diverse gender groups.

## Supplementary Information

Supplementary Material 1.

## Data Availability

The data that support the findings of this study are not publicly available due to restrictions based on the informed consent provided by the participants. However, the data are available from Sophie Baumann (sophie.baumann@med.uni-greifswald.de) upon reasonable request, in accordance with ethical and legal requirements. The input codes used for the Mplus analyses are provided in the appendix.

## Abbreviations

MHI-5: Mental Health Inventory - 5
CFA: Confirmatory factor analysis
WLSMV: Weighted Least Squares Mean and Variance adjusted
CFI: Comparative Fit Index
TLI: Tucker-Lewis Index
RMSEA: Root and Mean Square Error of Approximation
SRMR: Standardized Root Mean Square Residual
SD: Standard deviation
PHQ-9: Patient Health Questionnaire - 9
GAD-7: Generalized Anxiety Disorder - 7
AUDIT-C: Alcohol Use Disorders Identification Test - Consumption
SEP: Socio-economic position
