# Supplementary material for: Assessing evidence of measurement invariance of the Mental Health Inventory (MHI-5) by gender in a German adult sample
Source: BMC Psychiatry. 2025 Oct 2;25:917. doi: 10.1186/s12888-025-07463-2 (PMC12490041; doi:10.1186/s12888-025-07463-2)
Supplement: Supplementary file 1 — Supplementary Material 1. [file 12888_2025_7463_MOESM1_ESM.pdf]

Appendix:

MPlus Input code:

One-factor model, without grouping:

```
Title:
  1-Faktor Modell, ohne Gruppen

Data:
  File is PRINT_MHI.dat ;

Variable:
  Names are
     amhi1 amhi2 amhi3 amhi4 amhi5 ;

  Missing are all (-9999) ;

  Usevariables= amhi1 amhi2 amhi3 amhi4 amhi5 ;

  Categorical = amhi1 amhi2 amhi3 amhi4 amhi5 ;


Analysis:
  Estimator = WLSMV ; ! Kategoriale Daten

  Parameterization = theta; !für residual variances

  Starts=100;

Model:

F1 by amhi1* amhi2* amhi3* amhi4* amhi5*;

F1@1; !Varianz = 1 zur Identifikation

[amhi1$1-amhi1$4*]; !Thresholds sollen frei geschätzt werden
[amhi2$1-amhi2$4*]; !gleiches Resultat wenn ich das weglasse
[amhi3$1-amhi3$4*];
[amhi4$1-amhi4$4*];
[amhi5$1-amhi5$4*];

[F1@0]; !Factor means = 0 zur Identifikation

amhi1@1 amhi2@1 amhi3@1 amhi4@1 amhi5@1; !Residual variances auf 1 fixiert

Output:

  STDYX;
  tech4 ;
  standardized residual modindices(1.96);
```

Two-factor model, without grouping:

```
Title:
  2-Faktor Modell, ohne Gruppen
Data:
  File is PRINT_MHI.dat ;

Variable:
  Names are amhi1 amhi2 amhi3 amhi4 amhi5 ;

  Missing are all (-9999) ;

  Usevariables= amhi1 amhi2 amhi3 amhi4 amhi5 ;

  Categorical = amhi1 amhi2 amhi3 amhi4 amhi5 ;


Analysis:
  Estimator = WLSMV ; !weighted least squaremeter and variance adjusted, for categorical

  Parameterization = theta; !wichtig für residual variances, categorical


Model:
  F1 by amhi1* amhi3* amhi5*;!Stern 1. Faktorladung wird auch frei geschätzt
  F2 by amhi2* amhi4*;

  F1@1; !Varianz = 1 zur Identifikation
  F2@1;

[amhi1$1-amhi1$4*]; !Thresholds sollen frei geschätzt werden
[amhi2$1-amhi2$4*]; !gleiches Resultat wenn ich das weglasse
[amhi3$1-amhi3$4*];
[amhi4$1-amhi4$4*];
[amhi5$1-amhi5$4*];


[F1@0]; !Factor means = 0 zur Identifikation
[F2@0];

amhi1@1 amhi2@1 amhi3@1 amhi4@1 amhi5@1; !Residual variances auf 1 fixiert

Output:
 TECH4 ! Angaben über Parameterspezifikationen und Startwerte
 STDYX ! completely standardized
```

Two-factor model, women only:

```
Title:
  2-Faktormodell, nur Frauen ( = Referenzmodell)

Data:
  File is PRINT_MHI.dat ;

Variable:
  Names are amhi1 amhi2 amhi3 amhi4 amhi5 asex;

  Missing are all (-9999) ;

  Usevariables= amhi1 amhi2 amhi3 amhi4 amhi5 ;

  Categorical = amhi1 amhi2 amhi3 amhi4 amhi5 ;

  Useobservations = asex eq 0; !nur Frauen

Analysis:
  Estimator = WLSMV ; !weighted least squaremeter and variance adjusted, for categorical

  Parameterization = theta; !wichtig für residual variances, categorical

Model:
  F1 by amhi1* amhi3* amhi5*;!Stern 1. Faktorladung wird auch frei geschätzt
  F2 by amhi2* amhi4*;

  F1@1; !Varianz = 1 zur Identifikation
  F2@1;

[amhi1$1-amhi1$4*]; !Thresholds sollen frei geschätzt werden
[amhi2$1-amhi2$4*]; !gleiches Resultat wenn ich das weglasse
[amhi3$1-amhi3$4*];
[amhi4$1-amhi4$4*];
[amhi5$1-amhi5$4*];


[F1@0]; !Factor means = 0 zur Identifikation
[F2@0];

amhi1@1 amhi2@1 amhi3@1 amhi4@1 amhi5@1; !Residual variances auf 1 fixiert

Output:
 STDYX ! completely standardized
```

Two-factor model, men only:

```
Title:
  2-Faktormodell, nur Männer

Data:
  File is PRINT_MHI.dat ;

Variable:
  Names are amhi1 amhi2 amhi3 amhi4 amhi5 asex;

  Missing are all (-9999) ;

  Usevariables= amhi1 amhi2 amhi3 amhi4 amhi5 ;

  Categorical = amhi1 amhi2 amhi3 amhi4 amhi5 ;

  Useobservations = asex eq 1; !nur Männer

Analysis:
  Estimator = WLSMV ; !weighted least squaremeter and variance adjusted, for categorical

  Parameterization = theta; !wichtig für residual variances, categorical

Model:
  F1 by amhi1* amhi3* amhi5*;!Stern 1. Faktorladung wird auch frei geschätzt
  F2 by amhi2* amhi4*;

  F1@1; !Varianz = 1 zur Identifikation
  F2@1;

[amhi1$1-amhi1$4*]; !Thresholds sollen frei geschätzt werden
[amhi2$1-amhi2$4*]; !gleiches Resultat wenn ich das weglasse
[amhi3$1-amhi3$4*];
[amhi4$1-amhi4$4*];
[amhi5$1-amhi5$4*];


[F1@0]; !Factor means = 0 zur Identifikation
[F2@0];

amhi1@1 amhi2@1 amhi3@1 amhi4@1 amhi5@1; !Residual variances auf 1 fixiert

Output:
 STDYX ! completely standardized
```

Two-factor baseline model:

```
Title:
   CFA with grouping, baseline modell = Konfigurale Invarianz, 2 Faktoren

 Data:
   File is PRINT_MHI.dat ;

 Variable:
   Names are
      amhi1 amhi2 amhi3 amhi4 amhi5 asex;

   Missing are all (-9999) ;

   Usevariables= amhi1 amhi2 amhi3 amhi4 amhi5 asex;
   Categorical = amhi1 amhi2 amhi3 amhi4 amhi5 ;
   Grouping = asex (0=female, 1=male);
   Useobservations = 0 or 1;

 Analysis:
   Estimator = WLSMV ; ! Kategoriale Daten
   H1Iterations  = 3000;
   Starts=100;

 Model:

   ! Jede beobachtete kategoriale Variable bekommt eine latente Antwortvariable y*
   (Phantomvariable)
   y1 BY amhi1@1;
   y2 BY amhi2@1;
   y3 BY amhi3@1;
   y4 BY amhi4@1;
   y5 BY amhi5@1;

   ! Faktor 1: positive Items
   F1 BY y1* y3* y5*;

   ! Faktor 2: negative Items
   F2 BY y2* y4*;

   ! Faktor-Varianzen für Identifikation
   F1@1;
   F2@1;

   ! Mittelwerte fixieren
   [F1@0];
   [F2@0];

   ! Skalenfixierung der beobachteten Items
   {amhi1@1 amhi2@1 amhi3@1 amhi4@1 amhi5@1};
```

```
 ! Intercepts der Phantomvariablen auf 0
 [y1-y5@0];

 ! Residualvarianzen der Phantomvariablen auf 0 (sie sind vollständig durch amhi erklär
 y1-y5@0;

 ! Thresholds der beobachteten ordinalen Items frei schätzen
 [amhi1$1-amhi1$4*];
 [amhi2$1-amhi2$4*];
 [amhi3$1-amhi3$4*];
 [amhi4$1-amhi4$4*];
 [amhi5$1-amhi5$4*];

MODEL male:
 ! Gleiche Struktur für Männer

 F1 BY y1* y3* y5*;
 F2 BY y2* y4*;

 F1@1;
 F2@1;

 [F1@0];
 [F2@0];

 {amhi1@1 amhi2@1 amhi3@1 amhi4@1 amhi5@1};

 [y1-y5@0];

 y1-y5@0;

 [amhi1$1-amhi1$4*];
 [amhi2$1-amhi2$4*];
 [amhi3$1-amhi3$4*];
 [amhi4$1-amhi4$4*];
 [amhi5$1-amhi5$4*];

 OUTPUT:
 tech4;
 STDYX;

 SAVEDATA:
 DIFFTEST = mhi5_1.dat;
```

Two-factor, threshold invariance model:

```
Title:
  CFA with grouping, Threshold Invarianz, 2 Faktoren

Data:
  File is PRINT_MHI.dat ;

Variable:
  Names are
     amhi1 amhi2 amhi3 amhi4 amhi5 asex;

  Missing are all (-9999) ;

  Usevariables= amhi1 amhi2 amhi3 amhi4 amhi5 asex;
  Categorical = amhi1 amhi2 amhi3 amhi4 amhi5 ;
  Grouping = asex (0=female, 1=male);
  Useobservations = 0 or 1;

Analysis:
  Estimator = WLSMV ; ! Kategoriale Daten
  H1Iterations  = 3000;
  DIFFTEST = mhi5_1.dat;
  Starts=100;

Model:

  ! Jede beobachtete kategoriale Variable bekommt eine latente Antwortvariable y*
  y1 BY amhi1@1;
  y2 BY amhi2@1;
  y3 BY amhi3@1;
  y4 BY amhi4@1;
  y5 BY amhi5@1;

  ! Faktor 1: positive Items
  F1 BY y1* y3* y5*;

  ! Faktor 2: negative Items
  F2 BY y2* y4*;

  ! Faktor-Varianzen für Identifikation
  F1@1;
  F2@1;

  ! Mittelwerte fixieren
  [F1@0];
  [F2@0];

  ! Skalenfixierung der beobachteten Items in Gruppe 1, frei in Gruppe 2
  {amhi1@1 amhi2@1 amhi3@1 amhi4@1 amhi5@1};
```

```
! Intercepts der Phantomvariablen auf 0 in Gruppe 1, frei in Gruppe 2
[y1-y5@0];

! Residualvarianzen der Phantomvariablen auf 0 (sie sind vollständig durch amhi erklärt)
y1-y5@0;

! Thresholds sollen gleichgesetzt sein
[amhi1$1-amhi1$4*] (T1-T4);
[amhi2$1-amhi2$4*] (T5-T8);
[amhi3$1-amhi3$4*] (T9-T12);
[amhi4$1-amhi4$4*] (T13-T16);
[amhi5$1-amhi5$4*] (T17-T20);

MODEL male:

F1 BY y1* y3* y5*;
F2 BY y2* y4*;

F1@1;
F2@1;

[F1@0];
[F2@0];

{amhi1* amhi2* amhi3* amhi4* amhi5*};

[y1-y5*];

y1-y5@0;

[amhi1$1-amhi1$4*] (T1-T4);
[amhi2$1-amhi2$4*] (T5-T8);
[amhi3$1-amhi3$4*] (T9-T12);
[amhi4$1-amhi4$4*] (T13-T16);
[amhi5$1-amhi5$4*] (T17-T20);

OUTPUT:
tech1 tech4;
STDYX;

SAVEDATA:
DIFFTEST = mhi5_2.dat;
```

Two-factor, threshold and loading invariance:

```
Title:
  CFA with grouping, Loading + Threshold Invarianz, 2 Faktoren

Data:
  File is PRINT_MHI.dat ;

Variable:
  Names are
    amhi1 amhi2 amhi3 amhi4 amhi5 asex;

  Missing are all (-9999) ;

  Usevariables= amhi1 amhi2 amhi3 amhi4 amhi5 asex;
  Categorical = amhi1 amhi2 amhi3 amhi4 amhi5 ;
  Grouping = asex (0=female, 1=male);
  Useobservations = 0 or 1;

Analysis:
  Estimator = WLSMV ; ! Kategoriale Daten
  H1Iterations  = 3000;
DIFFTEST = mhi5_2.dat;
Starts=100;

Model:

  ! Jede beobachtete kategoriale Variable bekommt eine latente Antwortvariable y*
  y1 BY amhi1@1;
  y2 BY amhi2@1;
  y3 BY amhi3@1;
  y4 BY amhi4@1;
  y5 BY amhi5@1;

  ! Faktor 1: positive Items
  F1 BY y1* y3* y5* (L1-L3);

  ! Faktor 2: negative Items
  F2 BY y2* y4* (L4-L5);

  ! Faktor-Varianzen für Identifikation
  F1@1;
  F2@1;

  ! Mittelwerte fixieren
  [F1@0];
  [F2@0];

  ! Skalenfixierung der beobachteten Items in Gruppe 1, frei in Gruppe 2
  {amhi1@1 amhi2@1 amhi3@1 amhi4@1 amhi5@1};
```

```
  ! Intercepts der Phantomvariablen auf 0 in Gruppe 1, frei in Gruppe 2
  [y1-y5@1];

  ! Residualvarianzen der Phantomvariablen auf 0 (sie sind vollständig durch amhi erklärt)
  y1-y5@0;

  ! Thresholds sollen gleichgesetzt sein
  [amhi1$1-amhi1$4*] (T1-T4);
  [amhi2$1-amhi2$4*] (T5-T8);
  [amhi3$1-amhi3$4*] (T9-T12);
  [amhi4$1-amhi4$4*] (T13-T16);
  [amhi5$1-amhi5$4*] (T17-T20);

MODEL male:

  F1 BY y1* y3* y5* (L1-L3);
  F2 BY y2* y4* (L4-L5);

  F1*;
  F2*;

  [F1@0];
  [F2@0];

  {amhi1* amhi2* amhi3* amhi4* amhi5*};

 [y1-y5*];

  y1-y5@0;

  [amhi1$1-amhi1$4*] (T1-T4);
  [amhi2$1-amhi2$4*] (T5-T8);
  [amhi3$1-amhi3$4*] (T9-T12);
  [amhi4$1-amhi4$4*] (T13-T16);
  [amhi5$1-amhi5$4*] (T17-T20);

  OUTPUT:
  tech1 tech4;

  SAVEDATA:
  DIFFTEST = mhi5_3.dat;
```
